# Identification of susceptible HLA class II co-amoxiclav genotypes based on the analysis of drug-specific T-cells from patients with liver injury

**DOI:** 10.1186/2045-7022-4-S3-O3

**Published:** 2014-07-18

**Authors:** Katy Saide, Seung-Hyun Kim, Ye Yuan, Ann Daly, Ana Alfirevic, Munir Pirmohamed, Kevin Park, Dean Naisbitt

**Affiliations:** 1 MRC Centre for Drug Safety Science, University of Liverpool, Liverpool, UK; 2 Department of Allergy and Clinical Immunology, Ajou University School of Medicine, South Korea; 3 Institute of Cellular Medicine, Newcastle University, Newcastle, UK; 4 Department of Molecular and Clinical Pharmacology, University of Liverpool, Liverpool, UK

## 

Drug-induced liver injury (DILI) is a major concern for public health. Several forms of DILI are associated with the expression of specific MHC genes suggesting that the adaptive immune system is involved in the disease pathogenesis. We have identified drug-responsive T-cells in patients with flucloxacillin induced DILI and shown that the drug antigen is presented to T-cells in the context of the HLA risk allele (HLA-B*5701). The combination of amoxicillin and clavulanic acid (co-amoxiclav) is one of the most frequently prescribed drugs. Occasionally, human exposure is associated with DILI. Individual susceptibility can be influenced by HLA genotypes (e.g. DRB1*1501 risk; DRB1*07:01 risk) and we have recently characterized drug-specific T-cells in patients with co-amoxiclav-induced DILI. The objective of this study was to use amoxicillin-specific CD4+ T-cell clones from 2 DILI patients to investigate the mechanisms of drug antigen presentation and to identify susceptible HLA class II genotypes based on analysis of drug specific T-cell responses.

## Method

Antigen presenting cells (APC) expressing different HLA alleles generated from our HLA-typed PBMC cell bank were used to investigate the latter. EBV-transformed B-cell lines from 15 donors expressing HLA alleles of interest were generated and used as APC. To characterize mechanism(s) of T-cell activation, APC were (1) omitted from the assay, (2) pulsed with amoxicillin for 1-16h and (3) treated with MHC blocking antibodies.

## Results

The clones showed a strong proliferative response and IFN secretion following exposure to amoxicillin and autologous APC. Activation of the clones was also observed with APC pulsed with amoxicillin for 16h. The response was strongly inhibited with an anti-HLA class II antibody. More detailed analysis using HLA-DR, -DP and -DQ antibodies revealed that the response was HLA-DR restricted. Allogeneic APC were then used to define HLA-DR alleles involved in the drug-specific response. Clones were activated with amoxicillin and allogeneic APC expressing a range of DRB1 alleles including 03:01, 04:01, 13:01 and 15:01. In contrast, amoxicillin did not activate the clones with APC expressing HLA-DRB1*01:01 and 07:01.

## Conclusion

These findings characterizing HLA-restricted amoxicillin-specific T-cell responses agree with recent genome-wide association studies which identify specific HLA-DRB1 alleles as risk factors that influence susceptibility to co-amoxiclav-induced liver injury.

